# Potential and limitations of computed tomography images as predictors of the outcome of ischemic stroke events: a review

**DOI:** 10.3389/fstro.2023.1242901

**Published:** 2023-09-07

**Authors:** Gonçalo Oliveira, Ana Catarina Fonseca, José M. Ferro, Arlindo L. Oliveira

**Affiliations:** ^1^NeuralShift, Lisbon, Portugal; ^2^INESC-ID, Instituto Superior Técnico, Lisbon, Portugal; ^3^Faculdade de Medicina, Universidade de Lisboa, Lisbon, Portugal

**Keywords:** ischemic stroke, functional outcome, mRS, systematic review, machine learning, deep learning

## Abstract

The prediction of functional outcome after a stroke remains a relevant, open problem. In this article, we present a systematic review of approaches that have been proposed to predict the most likely functional outcome of ischemic stroke patients, as measured by the modified Rankin scale. Different methods use a variety of clinical information and features extracted from brain computed tomography (CT) scans, usually obtained at the time of hospital admission. Most studies have concluded that CT data contains useful information, but the use of this information by models does not always translate into statistically significant improvements in the quality of the predictions.

## 1. Introduction

After a stroke occurs, fast patient care is of paramount importance, given the rapid degradation of the patient's brain (Saver, 2006). In order to guide clinicians on what may be the best treatment to apply, the expected functional outcome of the patient is often considered (something the patients and their relatives are also interested in knowing). The most commonly used metric to assess this outcome is the modified Rankin scale (mRS). It is an integer scale that goes from 0 to 6, where the lower end corresponds to full independence and the upper end corresponds to death (Swieten et al., 1988).

Studies exploring the prediction of this variable can be categorized into three groups based on the information they consider: tabular approaches that rely solely on demographic and clinical variables, imaging-only approaches that exclusively utilize brain images obtained from imaging protocols, and hybrid approaches that incorporate both tabular and imaging data. This review aims to assess the potential value of imaging data in this prediction task, focusing primarily on the imaging-only and hybrid approaches. These approaches are generally less prevalent in the literature compared to the tabular approach. Specifically, we concentrate on studies utilizing brain computed tomography (CT) scans, including non-contrast CT scans (NCCT), which are the recommended initial scan procedure for stroke investigation due to their availability, speed, and patient tolerance (Hopyan et al., 2010). Additionally, we consider variants of CT scans that use contrast agents: CT angiography (CTA) and CT perfusion (CTP).

To this end, we searched PubMed using the following query: (“machine learning” OR “neural networks” OR “deep learning”) AND “stroke” AND (“prognosis” OR “prediction”). The resulting 719 articles were then filtered using the Rayyan collaborative tool (Ouzzani et al., 2016) in a blind process based on their title and, in case of doubt, their abstract as well. A paper was considered relevant if it focused on predicting the mRS variable using imaging data from CT scans. The papers deemed relevant by at least two reviewers were chosen for further analysis.

Regarding the exclusion criterion, we excluded studies that violated the following constraints. Studies analyzing other modalities besides CT as it would not be possible to analyse the CT influence individually, in such studies. Studies with missing patient data information and where such information was not possible to obtain from the authors. Studies not presenting original work, which also excludes reviews, meta-analyses, and editorials. Studies that were only focused on interventions or treatments without a direct relevance to predictive modeling using CT images. Studies not written in English or a language familiar to the research team.

**Figure 1:**
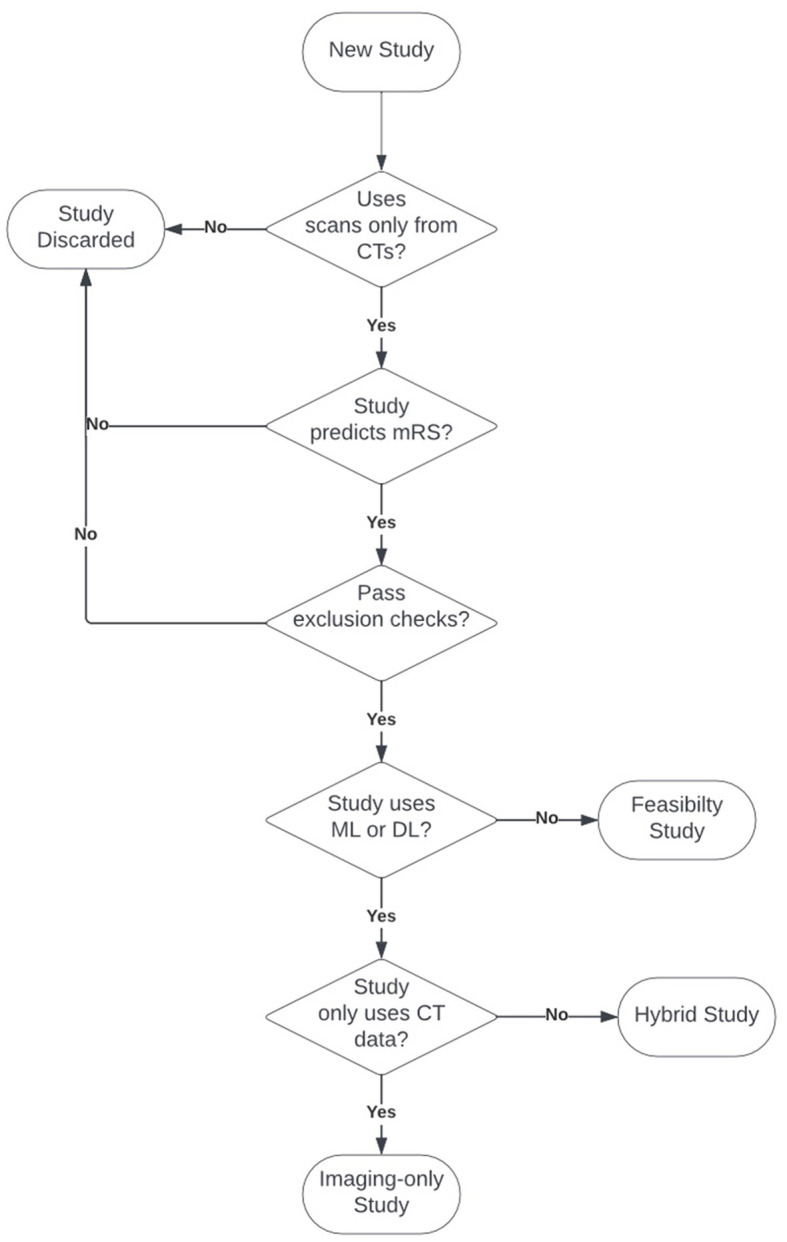
Studies inclusion criteria and classification flowchart.

The entire process, illustrated in Figure 1, led to the selection of 19 studies, which are summarized in Table 1. Among these studies, there were three feasibility analyses, four imaging-only studies, and 12 hybrid studies.

**Table 1:** Tabular summary of the studies considered in this review, ordered by date and then by name.

**References**	**Paper type**	**Cohort size**	**Good/bad outcome (%)**	**mRS time**	**mRS split**	**Dataset split**	**Modalities**	**Best AUC**	**Pre-processing steps**	**Adding imaging data improves?**	**Stroke type**	**Feature extraction method**	**Top 3 features**
Tong et al. (2017)	Hybrid	135	48/52	90 days	2	No mention	CTA, CTP, NCCT	0.85	No mention	Inconclusive	ACA stroke, AIS	Custom, Manual	No mention
Hilbert et al. (2019)	Image-only	1,301	36/64	90 days	2	4 fold CV	CTA	0.71	MIP, registration, skull stripping, windowing	N/A	AIS	DL	N/A
Nagel et al. (2019)	Study	388	34/56	45, 90 and 120 days	2	N/A	NCCT	N/A	Gantry tilt, registration	Potentially yes	AIS	e-Stroke	AAIV, e-ASPECTS
Xie et al. (2019)	Hybrid	512	47/53	90 days	0–5	Stratified 5 fold CV	CTA, CTP, NCCT	0.87	No mention	Inconclusive	AIS	Manual	Age, Baseline NIHSS, ASPECTS
Bacchi et al. (2020)	Hybrid	204	55/45	90 days	1	Random 85% train/ 15% test split; train used 10 fold CV	NCCT	0.75	N/A	Potentially yes	AIS	DL	No mention
Brugnara et al. (2020)	Hybrid	246	33/67	90 days	2	0.632 bootstrapping	CTA, CTP, NCCT	0.86	No mention	Inconclusive	ACA stroke, AIS, LVO, EVT	Manual, e-stroke	24 h NIHSS, premorbid mRS, final infarction volume
Mah et al. (2020)	Hybrid	1,696	45/55	Discharge	2	10 fold CV	NCCT	0.76	Registration, skull stripping	No	AIS	Custom	No mention
Samak et al. (2020)	Hybrid	500	25/75	90 days	2	80% train/ 20% test	NCCT	0.75	Windowing, Z-scaling	Inconclusive	AIS, LVO, EVT	DL	No mention
Brugnara et al. (2022)	Hybrid	1,103	31/69	90 days	2	0.632 bootstrapping	NCCT	0.85	Gantry tilt, registration	No	AIS, MCA stroke, EVT	Manual, e-ASPECTS	Premorbid mRS, baseline NIHSS, age
Cao et al. (2022)	Study	870	N/A	90 days	N/A	694 train/176 test	NCCT	N/A	Registration, skull stripping	Potentially yes	AIS	DL, Radiomics	No mention
Danala et al. (2022)	Image-only	31	52/48	After EMT	3	Leave-one-case-out evaluation	CTP	0.88	Skull stripping	N/A	AIS, LVO	Custom	N/A
Fang et al. (2022)	Image-only	31	50/50	Discharge	2	5-fold CV	NCCT	0.74	N/A	N/A	PCA Stroke	DL	N/A
Jabal et al. (2022)	Hybrid	293	34/66	90 days	2	Random spliting 75% train/ 25% test; train used 10 fold CV	CTA, NCCT	0.84	No mention	Potentially yes	AIS, EVT	e-Stroke	N/A
Kis et al. (2022)	Study	295	35/65	30 and 90 days	2	N/A	NCCT	N/A	No mention	Potentially yes	AIS, EVT	e-Stroke	N/A
Kniep et al. (2022)	Hybrid	149	31/69	90 days	2-5	Nested 5 fold CV	NCCT	0.90	Registration	Potentially yes	AIS, PCA Stroke	Radiomics	Cerebellum, midbrain, thalamus
Meng et al. (2022)	Hybrid	323	32/68	90 days	2	80% train/ 20% test	CTA	0.82	Windowing	Inconclusive	LVO, EVT	DL	No mention
Ramos et al. (2022)	Hybrid	3,279	38/62	90 days	2	5 fold CV with 80% train/20% validation in the training sets	CTA	0.81	Registration, skull stripping	No	AIS, LVO, EVT	DL, Radiomics	Age, baseline NIHSS, pre-stroke mRS
Samak et al. (2022)	Image-only	500	25/75	90 days	2	Stratified 70% train/ 15% val/ 15% test	NCCT	0.79	Registration, skull stripping, Z-scaling	N/A	AIS	DL	N/A
Ozkara et al. (2023)	Hybrid	185	54/46	90 days	2	60% train/20% val/20% test	CTA, CTP, NCCT	0.96	No mention	No mention	AIS, MCA stroke	Manual, RAPID	Discharge NIHSS, discharge BUN, age

The AUC scores are rounded to two decimal places. The acronyms BUN, AIS, EVT, EMT, and AAIV mean blood urea nitrogen, acute ischemic stroke, endovascular treatment, endovascular mechanical thrombectomy and automatically derived acute ischemic volume, respectively. N/A, not applicable.

## 2. Characterization of the studies

Regarding CT modalities, of the 19 studies examined, 15 included at least a NCCT scan and 10 used just this modality. All the imaging-only methods used only one modality (three with NCCT, one with CTA and one with CTP). Conversely, among the hybrid models, five out of 12 studies utilized more than one modality, and in all of these hybrid approaches, NCCT scans were consistently included.

In 17 of the studies, the primary focus was on predicting the mRS based on data available during the acute phase of stroke. However, two other studies (Fang et al., 2022; Meng et al., 2022) did not explicitly mention the phase from which their data was obtained.

Some of the studies only considered a specific type of stroke patients. For example, seven of them only considered patients selected for thrombectomy (EVT). Furthermore, six studies limited their analysis to groups of patients who experienced a stroke in particular arterial territories, with two studies focusing on the middle cerebral artery (MCA), two on the anterior cerebral artery (ACA), and two on the posterior cerebral artery (PCA).

Although the mRS is a seven point scale, it is rarely considered in its entirety. Instead, authors frequently split the scale into “good” and “poor” output at different mRS thresholds, simplifying the task to a binary classification problem. Considering that good outcome patients have an mRS ≤ 2 (and poor outcome patients have mRS > 2) is by far the most frequent strategy, with 16 of the studies using it. The exceptions were Bacchi et al. (2020) using a split at 1, Danala et al. (2022) using a split at 3 and Cao et al. (2022) mentioning no split. Considering these splits, the binary distribution of outcomes was roughly evenly distributed (≤ 5% difference between classes) in seven studies, and in the other 11 poor outcome was the majority class.

The other important factor related to the target variable is when it is assessed. The 90 day mRS is the most frequent choice, being used in 16 works. The exceptions were Mah et al. (2020) and Fang et al. (2022) who considered the discharge mRS and Danala et al. (2022) who considered the mRS after EVT.

Regarding the pre-processing applied to the scans, determining the optimal trade-off between preprocessing and model invariance remains an open question. Out of the 19 studies reviewed, eight authors employed template registration, six used skull stripping, five applied both techniques, and six studies conducted no pre-processing at all. In the studies that mentioned no pre-processing, the scans were either manually inspected by experts or processed using proprietary algorithms such as e-Stroke (Brainomix Ltd, Oxford, United Kingdom) or RAPID (iSchemaView, Menlo Park, USA).

Throughout this review, we compare the performance of the algorithms using the AUC metric, as it is the most commonly used metric to evaluate the performance of the proposed methods.

## 3. Feasibility analyses

Three of the studies analyzed in this review do not propose a specific algorithm to predict the mRS of stroke patients. Instead, their focus is on assessing the feasibility of predicting this variable from CT scan data. This distinction is crucial because if there were no evidence that CT scans contained prognostically relevant information, attempting to use them for prediction purposes would not be justified.

Nagel et al. (2019) and Kis et al. (2022) used e-Strokes tools to estimate biomarkers like acute ischemic volume (AIV) and ASPECTS and related them to the mRS target, using statistical analysis. They both conclude that both these biomarkers have the potential to be good predictors of patient outcome. In Cao et al. (2022) work, a custom deep learning (DL) algorithm is proposed to predict the ASPECT score, instead of using e-Stroke. These authors also conclude that this biomarker has the potential to be an important prognostic variable. Although these studies only focused on NCCTs, the other modalities (CTAs and CTPs) are in principle, at least as informative, meaning they should also contain relevant prognostic information.

## 4. Imaging-only studies

Of the four imaging-only approaches examined, three used DL and one used a custom algorithm. The DL algorithms all got an AUC bellow 0.8. Hilbert et al. (2019) and Fang et al. (2022) both used 2D neural networks. The former transformed the 3D CT volume into a 2D projection using maximum intensity projection (MIP) (Fishman et al., 2006) and the latter worked at the axial slice level.

Using CTA scans in their model, Hilbert et al. (2019) employed the MIP method to highlight brain arteries in the axial plane. The resulting 2D MIP image was then fed to their model, a ResNet (He et al., 2016) adapted with receptive field neural networks (RFNNs) (Jacobsen et al., 2016), to avoid overfitting. This model outperformed two baseline classifiers trained with 20 radiological imaging biomarkers (annotated by experts). The authors noticed their model tends to focus on the occluded arteries (that appear to be missing in the scans) by inspecting its activation mappings.

A segmentation model for nine posterior circulation structures was developed by Fang et al. (2022). The ground truth masks for this model were annotated by a neurologist. The proportions of affected tissue in each region were then used as features for a outcome prediction model. The authors note that their approach (0.74 AUC) predicted the discharge mRS better than pc-ASPECTS semiquantitative scale (0.67 AUC).

On the other hand, Samak et al. (2022) used the whole 3D volume of NCCTs. Their feature matching auto-encoder (FeMA) model not only predicts the dichotomised mRS score but also outputs a 3D image with the predicted one week stroke evolution. The authors used one week follow up scans as ground truth to train this model and compared it with other generative models. The predicted follow up scans gives a qualitative result useful for physicians and is also used by the model to improve the mRS prediction.

Finally, Danala et al. (2022), created a custom algorithm that uses CTP scans. For each of the images the CTP captures over time, their algorithm counts the number of “blood pixels” in each brain hemisphere. From this analysis, it creates two blood flow curves that represent the blood flow over time in each of the hemispheres. The idea is that big differences in these curves may indicate the presence of major large vessel occlusions (LVO). Several different features were extracted from these curves and used to predict the patient outcome, using machine learning (ML) classifiers like k-nearest neighbors (KNN) and support vector machines (SVM). This method obtained an AUC of 0.878 ± 0.077, but it is worth noting that they only analyzed 31 patients and, as mentioned, it used an unconventional mRS target, making it hard to compare with other results.

## 5. Hybrid studies

There are two main ways of incorporating imaging information into the prediction models:

- *Using imaging biomarkers* (5/12 papers) which are distinct characteristics of the image recognized by experts (examples of biomarkers are the ASPECT score or the occlusion site). Of the five hybrid studies that used biomarkers, only one obtained them from experts annotations (Xie et al., 2019). Three were obtained in a semi-automatic (algorithmic labeling revised by humans) (Brugnara et al., 2020, 2022; Ozkara et al., 2023) and the other one in a fully automatic way (Jabal et al., 2022).
- *Using features extracted by algorithms* (7/12 papers). Here, these features can be generated by DL approaches (Bacchi et al., 2020; Samak et al., 2020; Meng et al., 2022; Ramos et al., 2022) or using more traditional methods, like radiomics (Kniep et al., 2022; Ramos et al., 2022) or other hand-crafted features (Tong et al., 2017; Mah et al., 2020).

### 5.1. Imaging biomarkers

Several different ML models at various different mRS dichotomisation thresholds were tried by Xie et al. (2019). Their models used demographic, NIHSS and biomarkers variables from NCCT, CTA, and CTP scans. They achieved 0.748 and 0.772 AUC when the imaging variables and NIHSS were obtained at baseline and 24h after stroke onset, respectively. These results suggest that more up-to-date variables are more informative. Using feature selection, the authors were able to improve their models performance to 0.772 and 0.884, respectively. This feature selection step is not only relevant for model performance improvements but also for making it more robust to clinical usage, as it is easier to obtain the necessary information from patients.

Another work that also tried to use imaging features collected at different points in time was Brugnara et al. (2020). They also observed that the 24 h features resulted in the model with the best performance—0.856 AUC, in their case. They noted that adding CTP features did not improve the predictive performance of their models, when starting with a baseline containing NCCT and CTA biomarkers. Of the three most importance features considered by their algorithms—24 h NIHSS, premorbid mRS and final infarction volume—only the last is an imaging biomarker. These two facts raise the question of the relevance of the CT imaging in the outcome prediction.

Indeed, in their more recent study, Brugnara et al. (2022) tried to answer this question in a more principled way, comparing models with and without imaging biomarkers—acute ischemic volumes (AIV) and ASPECTS, in this study—using statistical tests. They note that both variables are strong independent predictors of the target 90 day mRS. Despite that, their conclusion is that there is no clear advantage in adding either AIV or ASPECTS (nor both), to a purely tabular baseline with just demographic and clinical variables. While the ASPECTS procedure is an established method for analyzing early infarct signs, the authors explain that it may be an overly simplistic approach, something which can limit its predictive power. In particular, this score weights all its ten brain regions equally and is invariant to infarct volume. The authors point out that ASPECTS and AIV were highly correlated variables, which explains both why they produce such similar results when used independently for prediction, and why combining them does not improve predictive performance.

In the study proposed by Ozkara et al. (2023), the focus was MCA patients and the authors tried different ML models with access to the three different CT modalities. They were able to achieve an impressive 0.958 AUC, albeit using a smaller dataset compared with the previous studies and not using cross validation for model evaluation. Using SHAP (Lundberg and Lee, 2017), they noted that discharge NIHSS score, discharge blood urea nitrogen (BUN) and age were the top three most important features in their best model (notably, none of them being an imaging biomarker). The fact that they used variables at discharge time is probably what explains their higher AUC score.

### 5.2. Algorithmicly generated features

The use of DL with CT imaging to predict stroke outcome was pioneered Bacchi et al. (2020) work. They developed a “branched” network where one branch uses a custom 3D CNN to encode a NCCT scan and the other branch encodes a collection of clinical and demographic variables. Although it only got an AUC of 0.75, this network outperformed the other tabular-only and imaging-only approaches experimented by them.

A branched network was also used in Samak et al. (2020) study. Their model introduced several new improvements such as the use of data augmentations, a more thorough pre-processing, focal loss (Lin et al., 2017) to help with class imbalance and attention mechanisms (Hu et al., 2018). In their dataset, this network achieved 0.75 AUC but that was enough to beat Bacchi et al. (2020) network and also a baseline model that only used clinical metadata (including imaging biomarkers).

In Meng et al. (2022) study, a custom branched network was also employed and obtained an AUC of 0.82, but it differed from the previous articles in some aspects. Instead of two branches, this study utilized three branches in parallel. The first branch encoded CT scan information, the second branch encoded the location of occluded vessels, and the third branch encoded other demographic and clinical features. Additionally, unlike the previous studies that used NCCTs, Meng et al. utilized CTAs.

Statistical tests were employed to assess if automatically extracted imaging features, either from radiomics or DL, can improve the outcome predictions, in Ramos et al. (2022) study. Their experiments were comprehensive, testing several different ML algorithms for the radiomics approach and several training methodologies for a ResNet-10 encoder (He et al., 2016). They also compared models trained on any combination of tabular data, imaging biomarkers and radiomics or DL features. In the end, they reached an AUC of 0.81 and concluded that the inclusion of imaging features does not improve model performance. Notably, unlike the previously mentioned studies, Ramos et al. (2022) directly concatenate the (unencoded) tabular features with the features produced by the imaging encoders. Finally, their SHAP (Lundberg and Lee, 2017) analysis points to age, baseline NIHSS, and pre-stroke mRS being the most important features (again, none of them being a biomarker).

Radiomics were also used by Kniep et al. (2022) who focused on posterior circulation strokes. These authors first registered the patient's NCCT scans to a MNI 152 (Brett et al., 2002) template to then extract radiomic features from the different pc-ASPECTS regions. These features, when combined with other clinical data obtained a 0.9 AUC, with the cerebellum, midbrain and thalamus being among the most relevant regions for the prediction task.

A custom algorithm to analyse the blood flow in CTP scans was also created by Tong et al. (2017), like it was done by Danala et al. (2022). However, Tong et al. (2017) algorithm is only semi-automatic, requiring expert input to select a region in circle of Willis for each of the three main brain arteries (MCA, PCA, and ACA). Time intensity curves are computed for each of these regions. Time intensity curves are also extracted for each “vascular pixel” and they are assigned to the territory with which they have the most similar territory intensity curve. The amount of blood in a territory is given by the amount of pixels assigned to that territory. They assume that high collateral scores correspond to high PCA/ACA scores. This collateral score is then combined with other features, including ASPECTS (from NCCTs) and recanalization (from CTAs) in a ML model that achieved 0.85 AUC.

Finally, Mah et al. (2020) also tried different models with progressively more variables, starting from a baseline with no imaging data. Their imaging features were extracted by a custom algorithm developed by the authors to segment lesions in NCCTs. Despite achieving an AUC of 0.76, the model that included the imaging information did not perform significantly better than the models where imaging information was not included.

## 6. Discussion and conclusions

Regarding the scale used to evaluate functional outcome, it is important to remember that the mRS has “moderate variability” between experts, as Mah et al. (2020) mention. Additionally, the AUC is known to provide unreliable estimates, particularly in low sample size and class imbalanced regimes (Hanczar et al., 2010) (characteristics of some of the studies here analyzed). Therefore, the results should be accompanied with additional metrics like the sensitivity, specificity or *F*_1_-score [something some authors already do (Bacchi et al., 2020; Danala et al., 2022; Ramos et al., 2022; Samak et al., 2022)].

An overview on the use of DL applied to imaging methods on stroke patients was done by Zhu et al. (2022). On their section about outcome prediction, they note that, compared with tissue fate prediction, outcome prediction may be a more difficult task. Their reasoning is that the outcome is influenced by several factors like age and stroke treatment, that are not accessible just using images. Naturally, we see that in the literature the hybrid models that are complemented by these additional variables perform better than the imaging-only models.

These authors also note that because the outcome prediction is a classification task, it has inherently less supervision than other tasks that have slice or voxel level annotations, meaning larger training datasets are required. Indeed, many of the studies we analyzed mention lack of data as a limiting factor of their work (Bacchi et al., 2020; Danala et al., 2022; Jabal et al., 2022; Kniep et al., 2022).

Models using variables collected at 24 h or discharge exhibited the best results, potentially good enough for clinical practice. However, the evident problem with such models is that they can not be used at admission. At the time of hospital admission, the consensus among researchers is that CT imaging contains relevant prognostic information. Unfortunately, all the studies that check if there is a statistically significant performance boost in adding such information conclude that these hybrid models are no better than their counterparts without imaging data (Brugnara et al., 2022; Ramos et al., 2022). This is consistent with the fact that imaging features are not regularly among the top most relevant features of the hybrid models (Brugnara et al., 2022; Ramos et al., 2022; Ozkara et al., 2023).

Despite the extensive literature on mRS prediction, there are relatively few works that attempt to predict this variable using imaging data, as evidenced by the relatively small size of this review. This may suggest that the research topic is still underexplored or may not hold significant promise. However, this second possibility seems incompatible with the results of feasibility studies, which assert the presence of relevant information in imaging data. Nonetheless, these studies also indicate that not all imaging variables retain statistical significance in multivariate analysis. In other words, while imaging data is relevant, its contribution may be limited when combined with other clinical data, further supporting the observations made in the previous paragraph.

Another reason for the limited number of studies considered in this review is its relatively narrow focus solely on CT images, while disregarding other brain imaging techniques like magnetic resonance imaging (MRI) and angiograms. Both MRI and angiograms provide relevant diagnostic information, with MRIs detecting small infarcts shortly after stroke onset and angiograms being valuable for grading collateral flow (Vital, 1999; Kim et al., 2004; Fonseca and Ferro, 2021). Arguably, these modalities may offer even more informative insights than CTs, known to be less sensitive to acute ischemic signs (de Lucas et al., 2008), which might contribute to their underrepresentation in the literature. However, it is worth noting that MRIs and angiograms are generally less available and come with more patient constraints compared to CTs, potentially impeding the deployment of solutions based on them in clinical practice.

## Author contributions

GO, AF, and AO were involved in the selection of the studies. GO read and summarized them and compiled the main text. All authors helped write and review the final document.
