# Supplementary material for: Potential and limitations of computed tomography images as predictors of the outcome of ischemic stroke events: a review
Source: Front Stroke. 2023 Sep 7;2:1242901. doi: 10.3389/fstro.2023.1242901 (PMC12802751; doi:10.3389/fstro.2023.1242901)
Supplement: Supplementary file 1 [file Data_Sheet_1.PDF]

Name	Main Author	Year	Target	Paper Type	Patients	Best AUC	Good outcome (%)	Bad outcome (%)	Dataset split	Modality	mRS split	Pre-processing steps	Adding imaging significantly improves	Stroke Type	Feature Extraction Method	Important features
**FeMA: Feature matching auto-encoder for predicting ischaemic stroke evolution and treatment outcome**	Samak	2022	mRS at 90 days	Image-only	500	0.79	0.25	0.75	stratified 70% train/ 15% val/ 15% test	NCCT	mRS ≤ 2 / mRS > 2	Registration, Skull Stripping, Z-scaling	NA	AIS	DL	NA
**Quantifying the Impact of Chronic Ischemic Injury on Clinical Outcomes in Acute Stroke With Machine Learning**	Mah	2020	mRS at discharge	Hybrid	1696	0.76 (95% 0.74 – 0.78)	0.45	0.55	10 fold CV	NCCT	mRS ≤ 2 / mRS > 2	Registration, Skull Stripping	NO - The optimal model incorporated the clinical features, pre-mRS and imaging (in the form of the combined lesion mask and zeta map) achieving an AUROC of 0.76 (95% CI 0.74–0.78) (Figure 3B). However, it did not perform significantly better than the same model with imaging information excluded	AIS	Custom	No mention
**Data-efficient Deep Learning of Radiological Image Data for Outcome Prediction after Endovascular Treatment of Patients with Acute Ischemic Stroke**	Hilbert	2019	mRS at 90 days	Image-only	1301	0.71	0.36	0.64	4 fold CV	CTA	mRS ≤ 2 / mRS > 2	MIP, Registration, Skull Stripping, Windowing	NA	AIS	DL	NA
**Application of Deep Learning to Ischemic and Hemorrhagic Stroke Computed Tomography and Magnetic Resonance Imaging**	Zhu	2022		Survey		NA			NA				Automated imaging analysis might be helpful in the prediction of clinical outcome, but further study of such predictions is warranted.			NA
**Use of Gradient Boosting Machine Learning to Predict Patient Outcome in Acute Ischemic Stroke on the Basis of Imaging, Demographic, and Clinical Information**	Xie	2019	mRS at 90 days	Hybrid	512	0.873	0.47	0.53	Stratified 5 fold CV	CTA, CTP, NCCT	mRS ≤ 0 / mRS > 0, mRS ≤ 1 / mRS > 1, mRS ≤ 2 / mRS > 2, mRS ≤ 3 / mRS > 3, mRS ≤ 4 / mRS > 4, mRS ≤ 5 / mRS > 5	No mention	Inconclusive - 90-day mRS outcome is influenced by (…) the (…) ASPECTS score; and vascular biomarkers including the presence of HMCAS, the degree of occlusion, and the degree of cervical carotid stenosis.	AIS	Manual	ASPECTS, HMCAS presence, baseline TIMI score
**Interpretable Machine Learning Modeling for Ischemic Stroke Outcome Prediction**	Jabal	2022	mRS at 90 days	Hybrid	293	0.84	0.34	0.66	Random spliting 75% train/25% test; train used 10 fold CV	CTA, NCCT	mRS ≤ 2 / mRS > 2	No mention	Potentially yes - the most important features for the final model outcome prediction were (…) occlusion side, degree of brain atrophy (…) early ischemic core (…), and circulation deficit volume on CTA. This demonstrates the opportunity for (…) imaging modalities (CT and CTA) to improve the precision of patient profiling for AIS management.	AIS, Thrombectomy (EVT)	e-Stroke	occlusion side, degree of brain atrophy, ASPECTS, collateral circulation
**Automated quantification of atrophy and acute ischemic volume for outcome prediction in endovascular thrombectomy**	Kis	2022	mRS at 30 days, mRS at 90 days	Study	295	NA	0.35	0.65	NA	NCCT	mRS ≤ 2 / mRS > 2	No mention	Potentially yes - imaging markers of stroke severity (…) offer potentially useful information when predicting neurological improvement	AIS, Thrombectomy (EVT)	e-Stroke	NCCT AIV, e-ASPECTS, atrophy and TICI status
**Deep learning derived automated ASPECTS on non-contrast CT scans of acute ischemic stroke patients**	Cao	2022	mRS at 90 days	Study	870	NA			694 train / 176 test	NCCT		Registration, Skull Stripping	Potentially yes - we found that the ASPECTS score correlates with the CTP core volume and 90DmRS, which could be useful in assisting future clinic treatment and prognosis.	AIS	DL, Radiomics	No mention

**Prediction of Functional Outcome in Stroke Patients with Proximal Middle Cerebral Artery Occlusions Using Machine Learning Models**	Ozkara	2023	mRS at 90 days	Hybrid	185	0.958 AUC (CI = 0.886–1)	0.54	0.46	60% train / 20% val / 20% test	CTA, CTP, NCCT	mRS ≤ 2 / mRS > 2	No mention	No mention - but imaging features is not among the top predictors	AIS, MCA Stroke	Manual, RAPID	None of the variables are imaging biomarkers - discharge NIHSS score, discharge BUN (Blood urea nitrogen), age, age-related admission SI (Shock Index), and discharge WBC (White blood cell)
**Clinical value of automated volumetric quantification of early ischemic tissue changes on non-contrast CT**	Brugnara	2022	mRS at 90 days	Hybrid	1103	0.852 (95% CI 0.850-0855)	0.31	0.69	0.632 bootstrapping procedure for cross-validation	NCCT	mRS ≤ 2 / mRS > 2	Gantry Tilt, Registration	NO - No significant difference was found between machine-learning models using either AIV or ASPECTS or both metrics for predicting a good clinical outcome (p>0.05). Although, they are both good individual predictors of the mRS	AIS, MCA Stroke, Thrombectomy (EVT)	Manual, e-ASPECTS	The top feature by importance in all models was the premorbid mRS (…) The following top 3 features are: A - NIHSS, age, glucose. B - age, NIHSS, AIV. C - ASPECTS, NIHSS, age. D - ASPECTS, age, NIHSS
**Novel and Efficient Quantitative Posterior-Circulation-Structure-Based Scale via Noncontrast CT to Predict Ischemic Stroke Prognosis: A Retrospective Study**	Fang	2022	mRS at discharge	Image-only	31	0.74	0.5	0.5	5-fold CV	NCCT	mRS ≤ 2 / mRS > 2		NA	PCA Stroke	DL	NA
**Developing new quantitative CT image markers to predict prognosis of acute ischemic stroke patients**	Danala	2022	mRS after EMT	Image-only	31	0.878±0.077	0.52	0.48	Leave-one-case-out evaluation	CTP	mRS ≤ 3 / mRS > 3	Skull Stripping	NA	AIS, LVO	Custom	NA
**Time-resolved CT assessment of collaterals as imaging biomarkers to predict clinical outcomes in acute ischemic stroke**	Tong	2017	mRS at 90 days	Hybrid	135	0.85 (95% CI 0.78-0.91)	0.48	0.52	No mention	CTA, CTP, NCCT	mRS ≤ 2 / mRS > 2	No mention	Inconclusive - Collateral score is a prognostic biomarker among patients who achieved recanalization, but is not prognostic among patients who failed to achieve recanalization.	ACA Stroke, AIS	Custom, Manual	No mention
**Imaging-based outcome prediction in posterior circulation stroke**	Kniep	2022	mRS at 90 days	Hybrid	149	0.90 (95% CI 0.88-0.92)	0.31	0.69	nested 5 fold CV	NCCT	mRS ≤ 2 / mRS > 2, mRS ≤ 3 / mRS > 3, mRS ≤ 4 / mRS > 4, mRS ≤ 5 / mRS > 5	Registration	It is not compared with a clinical data only approach - The combined clinical data and machine learning-based model had the highest predictive performance with ROC-AUCs reaching 0.90 for mRS ≤ 2. (…) significant coefficients for pc-ASPECTS and NIHSS at admission (P-value < 0.05)	AIS, PCA Stroke	Radiomics	Feature importance analyses of the mean top 300 predictors of all training data sets show that pc-ASPECTS regions with the highest predictive power are cerebellum (30%), midbrain (29%) and thalamus (27%)

**e-ASPECTS derived acute ischemic volumes on non-contrast-enhanced computed tomography images**	Nagel	2019	mRS at 120 days, mRS at 45 days, mRS at 90 days	Study	388	NA	0.34	0.56	NA	NCCT	mRS ≤ 2 / mRS > 2	Gantry Tilt, Registration	Potentially yes - automatically derived acute ischemic volumes (AAIV) and e-ASPECTS are predictors of good outcome	AIS	e-Stroke	Within the whole cohort, the AAIV (OR 0.98 per ml, 95% CI 0.96–0.99) and e-ASPECTS scores (OR 1.3, 95%CI 1.07–1.57) were independent predictors of good outcome
**Multimodal Predictive Modeling of Endovascular Treatment Outcome for Acute Ischemic Stroke Using Machine-Learning**	Brugnara	2020	mRS at 90 days	Hybrid	246	0.856	0.33	0.67	0.632 bootstrapping procedure	CTA, CTP, NCCT	mRS ≤ 2 / mRS > 2	No mention	Inconclusive - CTP features did not help, “interventional characteristics” features did. The baseline model already has imaging features (but only got 0.74 AUC). There is no model with no imaging features to compare it with.	ACA Stroke, AIS, LVO, Thrombectomy (EVT)	Manual, e-Stroke	NIHSS after 24 hours, premorbid mRS and final infarction volume
**End-to-end artificial intelligence platform for the management of large vessel occlusions: A preliminary study**	Meng	2022	mRS at 90 days	Hybrid	323	0.82 (95% CI: 0.79-0.84)	0.32	0.68	training (80%) and test (20%) sets	CTA	mRS ≤ 2 / mRS > 2	Windowing	No comparison with baseline model w/o imaging features	LVO, Thrombectomy (EVT)	DL	No mention
**Deep Learning in the Prediction of Ischaemic Stroke Thrombolysis Functional Outcomes: A Pilot Study**	Bacchi	2020	mRS at 90 days	Hybrid	204	0.75	0.55	0.45	random 85% train/ 15% test split and 10 fold CV on the train set	NCCT	mRS ≤ 1 / mRS > 1		Potentially yes - The highest accuracy and AUC were achieved by the CNN + ANN combination utilizing both clinical data and (…) [imaging] data. Imaging-only was outperformed by clinical data only models	AIS	DL	No mention
**Prediction of Thrombectomy Functional Outcomes Using Multimodal Data**	Samak	2020	mRS at 90 days	Hybrid	500	0.75	0.25	0.75	80% train/ 20% test split	NCCT	mRS ≤ 2 / mRS > 2	Windowing, Z-scaling	Inconclusive - Their baseline “clinical data only” ANN also contains imaging biomarkers, so their work was not compared with any completely fair baseline. Their model that uses both imaging + clinical data had the best performance	AIS, LVO, Thrombectomy (EVT)	DL	No mention
**Combination of Radiological and Clinical Baseline Data for Outcome Prediction of Patients With an Acute Ischemic Stroke**	Ramos	2022	mRS at 90 days	Hybrid	3279	0.81	0.38	0.62	5 fold CV with 80% train/ 20% validation in the training sets	CTA	mRS ≤ 2 / mRS > 2	Registration, Skull Stripping	NO - the addition of image features does not improve the prediction. The performance of the imaging-only methods was worse than that of the clinical or hybrid models	AIS, LVO, Thrombectomy (EVT)	DL, Radiomics	Age, NIHSS at baseline, and pre-stroke mRS were found to be the top most important variables for functional outcome prediction. CRP (C-reactiveprotein) level at baseline + collateral score were the 2 other among the top 5
